# Sclerosing Polycystic Adenosis of the minor salivary gland: case report

**DOI:** 10.1590/S1808-86942010000200021

**Published:** 2015-10-19

**Authors:** Clarissa Araújo Silva Gurgel, Valéria Souza Freitas, Eduardo Antônio Gonçalves Ramos, Jean Nunes dos Santos

**Affiliations:** MSc., Substitute Professor; MSc., Assistant Professor; PhD, Adjunct Professor IV; PhD, Adjunct Professor IV

**Keywords:** salivary gland diseases, oral mucosa

## INTRODUCTION

Sclerosing Polycystic Adenosis (SPA) is a rare disease of the salivary glands, first described by Smith et al.^1^ in 1996, with histomorphological aspects similar to those from breast fibrocystic disorders1. Its pathogenesis is still unclear; however, it is considered a pseudo-neoplastic or reactive process^1^, ^2^, ^3^. Recently, Skálová et al.^4^ showed clonality in six SPA cases.

SPA affects mainly the parotid and submandibular glands, being much less frequent on the minor salivary glands^2^. Here, we report a case of SPA in a minor salivary gland and we discuss its histomorphological aspects.

## CASE PRESENTATION

**Figure 1:**
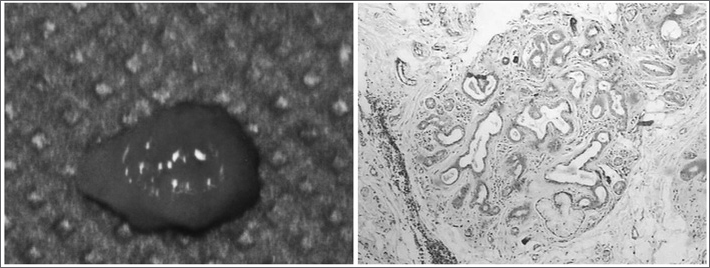
Sclerosing Polycystic Adenosis - Nodular aspect of the lesion after surgical excision. In the slide, notice a fibrous nodule permeated by interconnecting tubules, sometimes ectasic.

Female, African-Brazilian, 82 years old, came to us complaining of pain in the mouth floor when using her full mandible denture. During her intraoral exam, we noticed a misfitting of the upper and lower dentures and an ulcerated, reddish and firm nodule, measuring 2cm in its longest axis. The patient was instructed to refrain from using her dentures and after 15 days she returned for nodule reassessment - which remained unaltered. With clinical suspicion of an erythroplastic lesion, we carried out an incisional biopsy and the material was then referred to the Laboratory of Surgical Pathology. The lesion was characterized by fibrous nodules permeated by interconnecting tubules, either ectasic or with apocrine metaplastia (Figure 1). Some ductal elements were hyperplastic and there were atypic and vacuolated cells, followed by sparse mononuclear inflammatory infiltrate. Immunohistochemistry showed that the tubuloacinar elements which were positive to CKAE1/AE3, EMA, S-100, smooth muscle actin, GCDFP-15 and Ki-67 antibodies marked less than 1% of the cells. Estrogen, progesterone and CK 34βE12 were negative. SPA was diagnosed on the mouth floor, and the patient was later on submitted to total lesion exeresis. After 24 months there were no signs of recurrence.

## DISCUSSION

The literature has approximately 35 cases of SPA reported so far, with only four of those involving minor salivary glands. Therefore, this is the fifth case of minor salivary gland SPA.

Since this is a rarely described lesion, there is no characteristic clinical data for it. Smith et al.^1^ published a series of 9 cases, in which the average age of the patients affected by this problem was 28 years, with mild predilection for females. Gneep et al.^2^ reported an average age of 44.5 years and a mild predilection for men. An increase in nodular volume by slow growth, as we had in our case, is one of the most common clinical aspects^3^.

The histomorphological aspects hereby described fit those established for SPA. The lesion was made up of fibrous nodules, permeated by interconnecting tubules, either ectasic or with apocrine metaplasia, also showing cell vacuolization and atypia^1^, ^2^, ^3^^,^^5^. According to Cheuk, Chan^3^, SPA histological aspects are still not very known and, often times, such lesion is diagnosed as a malignant neoplasia, and a highlight among them is the ductal carcinoma^1^^,^^2^^,^^5^, cystic-adenocarcinoma, acinar cell carcinoma and mucoepidermoid carcinoma1. Sclerosing sialoadenites has also been included^1^^,^^2^, ^3^, ^4^, ^5^.

Immunohistochemistry for proteins S-100 and smooth muscle actin, from the case presented, showed the presence of myoepithelial cells in the tubule-acinar elements of the lesion^1^^,^^2^. The presence of such cells shows that despite the cell atypia seen in the SPA, the glandular ducts are functionally normal^2^. Gneep^5^ was also positive for cytokeratin and EMA in the ductal and acinar cells. Immune-staining for estrogen, progesterone and GCDGP-15 has also been described^3^^,^^5^^,^^6^, suggesting the participation of hormonal factors in the SPA^6^ pathogenesis. In this case, GCDFP-15 was positive; however, it is necessary to have the immunohistochemical study of other cases in order to establish the histogenetic profile of SPA.

SPA's treatment is based on lesion excision - with safe margins. Follow up must be careful because recurrences are common^2^^,^^6^.

## FINAL REMARKS

SPA's diagnosis must be carefully considered, especially because of its histomorphological similarity with different malignant neoplasias of the salivary gland.
